# Visceral fat area and blood lipids in colorectal cancer: predictors of surgical risk and prognosis

**DOI:** 10.3389/fonc.2026.1809966

**Published:** 2026-05-04

**Authors:** Yun Wang, Jun Bu, Dalin Xu, Chenyang Zhan, Jiaqi Hu, Minghao Zhang, Kejin Zhu, Yang Qi

**Affiliations:** 1 Department of General Surgery, Sichuan University Affiliated, Chengdu Second People’s Hospital, Chengdu, China; 2 Department of General Surgery, Chengdu Medical College, Chengdu, China; 3 Department of General Surgery, West China Hospital of Sichuan University, Chengdu, China; 4 Department of General Surgery, No. 363 Hospital, Chengdu, China; 5 Department of General Surgery, North Sichuan Medical College, Nanchong, China

**Keywords:** blood lipids, colorectal cancer, obesity paradox, prognosis, surgical risk, visceral fat area

## Abstract

**Objective:**

This study aimed to evaluate the predictive value of combining visceral fat area (VFA) with blood lipid levels for perioperative risk and long-term prognosis in patients undergoing radical resection for colorectal cancer (CRC).

**Methods:**

We conducted a retrospective analysis of 482 CRC patients who underwent radical surgery between December 2014 and December 2022. Patients were categorized into four groups based on VFA (visceral obesity defined as VFA ≥ 100 cm²) and lipid levels (elevated: TC ≥ 6.2 mmol/L, LDL-C ≥ 4.1 mmol/L, TG ≥ 2.3 mmol/L, or HDL-C < 1.0 mmol/L). Baseline characteristics, surgical outcomes, and survival data were compared. Multivariate logistic and Cox regression models were used to identify independent factors influencing complications and survival.

**Results:**

The combined VFA/lipid groups showed no significant differences in short-term complications (*P* > 0.05) but demonstrated significant stratification in long-term survival. Patients with both high VFA and high lipid levels (Group 2) exhibited significantly better overall survival (OS) and recurrence-free survival (RFS) compared to those with low VFA and normal lipids (HR ≈ 0.50, *P* < 0.01), supporting the “obesity paradox.” Age, TNM stage, and surgical approach were also identified as independent prognostic factors.

**Conclusion:**

The combination of VFA and blood lipid levels may serve as a useful preoperative indicator for risk stratification and prognostic assessment in CRC patients, with high VFA and high lipids potentially associated with improved survival outcomes.

## 1 Background

Colorectal cancer (CRC) remains the third most common malignancy worldwide, with surgical resection serving as the primary curative treatment (1). However, significant heterogeneity exists in postoperative complication rates (2) and long-term survival outcomes (3) among patients, underscoring the need for precise preoperative risk stratification and prognostic assessment. In recent years, the role of metabolic factors in cancer prognosis has garnered increasing attention (4, 5).

Obesity, particularly central obesity characterized by visceral fat accumulation, is thought to promote tumor progression through chronic inflammatory mechanisms (6, 7). Paradoxically, the “obesity paradox” (8) observed in patients with advanced cancer suggests that being overweight or mildly obese may be associated with improved survival, although the underlying mechanisms remain unclear.

Dyslipidemia, another important metabolic indicator, also exhibits a controversial relationship with CRC prognosis (9). Traditional metrics such as body mass index (BMI) are inadequate for accurately reflecting visceral fat distribution and metabolic abnormalities (10). Visceral fat area (VFA) (11), quantitatively assessed via CT imaging, provides a more metabolically relevant measure. Most current studies focus on single metabolic indicators, failing to comprehensively capture the complex metabolic status of patients. Therefore, this study innovatively combines VFA and blood lipid levels to address this research gap. We hypothesize that the combination of VFA and blood lipids can serve as an effective predictor of surgical risk and long-term prognosis in CRC patients, and that a “metabolic advantage” may exist in patients with both high VFA and high lipid levels, thereby improving survival outcomes.

## 2 Methods

### 2.1 Study population

#### 2.1.1 Patient enrollment

This retrospective study enrolled patients who underwent radical resection for colorectal cancer in the Department of General Surgery at Chengdu Second People’s Hospital between December 2014 and December 2022.

#### 2.1.2 Inclusion and exclusion criteria

##### 2.1.2.1 Inclusion criteria

(1) Underwent R0 radical resection (defined as complete macroscopic tumor removal with microscopically negative margins) at our institution; (2) Age ≥ 18 years; (3) Postoperative pathological confirmation of colorectal adenocarcinoma; (4) Preoperative abdominal CT examination performed within two weeks prior to surgery; (5) Complete medical records without missing clinical or laboratory data; (6) Agreement for the use of medical records for non-profit scientific research.

##### 2.1.2.2 Exclusion criteria

(1) Emergency surgery; (2) CT images with artifacts or noise preventing accurate software processing; (3) Presence of other concurrent malignant tumors; (4) Severe cardiovascular, pulmonary, cerebrovascular diseases, or other end-stage organ dysfunction; (5) Preoperative use of lipid-lowering medications, or a documented history of dyslipidemia with ongoing lipid-lowering treatment (to avoid pharmacologically altered lipid profiles); (6) Receipt of neoadjuvant radiotherapy or chemotherapy; (7) History of abdominal surgery; (8) Pre-existing severe autoimmune diseases; (9) Conditions affecting basal metabolism such as hyperthyroidism, hypothyroidism, or tuberculosis; (10) Uncontrolled psychiatric disorders or lack of full civil capacity; (11) Preoperative paralysis, long-term bedridden status, or inability to perform self-care.

#### 2.1.3 Sample size calculation

The sample size was determined *a priori* using a chi-square test for comparing multiple independent proportions (12), based on survival data from previous studies (7, 13–15). The significance level (α) was set at 0.05 and statistical power (1−β) at 0.8. With a λ value of 12.65 obtained from standard statistical tables, the initial calculation indicated that a total sample size of approximately 385 patients (around 97 per group) would be required. After accounting for an estimated 12% loss to follow-up, the minimum sample size was adjusted to 111 patients per group, yielding a total of at least 444 patients. The final cohort comprised 482 patients, adequately meeting the calculated sample size requirement.


$$n=\frac{\lambda }{\left(\sum_{i=1}^{k}\frac{{\left(p_{i}-\bar{p}\right)}^{2}}{\bar{p}}\right)}$$

n = required sample size per group.

λ = a constant depending on the significance level (α) and test power (1-β), obtained from tables.

k = number of groups (k=4 in this study).

pi = expected proportion for the i-th group (five-year survival rate).

p̄ = arithmetic mean of the proportions for all groups.

#### 2.1.4 Ethical approval

This study was approved by the Institutional Ethics Committee with a waiver of informed consent (Approval No: [KY]PJ224223). All data were de-identified and systematically collected using standardized methods.

### 2.2 CT imaging protocol and analysis

#### 2.2.1 CT scanning parameters

Abdominal CT scans were performed using a Siemens 256-slice computed tomography scanner (SENSATION 256; Siemens Healthineers, Germany). Scanning parameters were standardized as follows: tube voltage 120 kVp; automatic tube current modulation (100–250 mA); slice thickness 3 mm; reconstruction thickness 1 mm; pitch 0.8; matrix size 512×512. The scanning range extended from the xiphoid process to the symphysis pubis.

#### 2.2.2 Patient preparation

Patients fasted for 6 hours prior to scanning. During imaging, patients were placed in the supine position with arms raised above the head, and images were acquired during end-inspiration breath-hold to minimize motion artifacts.

### 2.3 CT image analysis

#### 2.3.1 CT image analysis and region of interest

##### 2.3.1.1 Delineation

**Figure 1:**
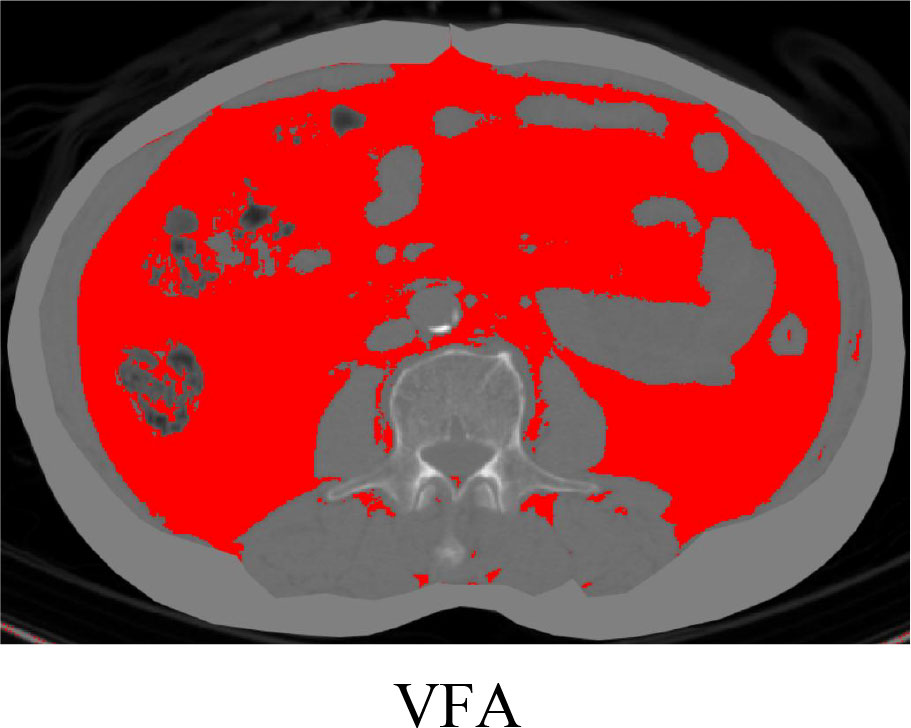
The above images show fat distribution at the level of the third lumbar vertebra (L3) displayed by ImageJ software.

We used ImageJ software (16)(National Institutes of Health, NIH, USA) to analyze axial CT images at the level of the L3 vertebral body. Tissue segmentation utilized Hounsfield Unit (HU) thresholds: visceral fat area (VFA) was defined as areas between -150 and -50 HU (17). Two independent radiologists, blinded to clinical outcomes, manually delineated the regions of interest (ROIs) for VFA. Inter-observer consistency was assessed using the intraclass correlation coefficient (ICC), with ICC >0.90 indicating high agreement (**Figure 1**).

#### 2.3.2 Data definitions and grouping

##### 2.3.2.1 Visceral obesity

Defined as VFA ≥100 cm² (18). Fasting blood samples were collected within one week preoperatively. All lipid measurements were performed in the same central laboratory using standard enzymatic methods with consistent quality control. Patients with acute infections, recent major surgery, or other acute stress conditions that could transiently alter lipid levels were not specifically excluded; however, such acute events were rare in this elective surgery cohort. To minimize variability, all samples were collected after an 8-hour overnight fast, within one week prior to surgery, and before any major physiological stress.

Dyslipidemia was defined according to the Chinese Adult Dyslipidemia Prevention Guidelines (19) as meeting any of the following: total cholesterol (TC) ≥ 6.2 mmol/L (≈240 mg/dL), low-density lipoprotein cholesterol (LDL-C) ≥ 4.1 mmol/L (≈160 mg/dL), triglycerides (TG) ≥ 2.3 mmol/L (≈200 mg/dL), or high-density lipoprotein cholesterol (HDL-C) < 1.0 mmol/L (≈40 mg/dL).

Based on these definitions, patients were categorized into four groups:

Group 1 (Low VFA/Normal Lipids): VFA < 100 cm², no dyslipidemia.

Group 2 (High VFA/Dyslipidemia): VFA ≥ 100 cm², with dyslipidemia.

Group 3 (High VFA/Normal Lipids): VFA ≥ 100 cm², no dyslipidemia.

Group 4 (Low VFA/Dyslipidemia): VFA < 100 cm², with dyslipidemia.

### 2.4 Data collection

Baseline and perioperative data were systematically collected, including: Demographics: age, sex, body mass index (BMI); Comorbidities: cardiovascular disease, chronic obstructive pulmonary disease (COPD), diabetes mellitus;Tumor characteristics: tumor location, histological grade, TNM stage;Perioperative variables: surgical type (e.g., laparoscopic vs. open), operation time, colostomy;Short-term outcomes: postoperative complications (non-infectious: anastomotic leakage, ileus, hypoproteinemia; infectious: wound infection, intra-abdominal infection, pneumonia, urinary tract infection), time to first flatus (FPT), and length of hospital stay (LOS).

### 2.5 Outcome measures

#### 2.5.1 Primary endpoint

Overall survival (OS), defined as the time from surgery to death from any cause or the last follow-up.

##### 2.5.1.1 Secondary endpoints

Recurrence-free survival (RFS), defined as the time from surgery to tumor recurrence, metastasis, or the last follow-up. The incidence of 30-day postoperative complications (as defined above).

Complications were recorded as binary outcomes (presence/absence) for each predefined category. Severity grading (e.g., Clavien-Dindo) was not applied, as our primary focus was on the occurrence of specific complication types.

### 2.6 Statistical analysis

Continuous variables were expressed as mean ± standard deviation or median (interquartile range) based on their distribution, and compared using Student’s t-test (normal distribution) or the Mann–Whitney U test (non-normal distribution). Categorical variables were presented as numbers (percentages) and analyzed using the chi-square test or Fisher’s exact test, as appropriate. For the identification of independent predictors, multivariable logistic regression (for postoperative complications) and Cox proportional hazards regression (for survival outcomes) were performed. To ensure comprehensive adjustment for potential confounders and to avoid the bias associated with data-driven variable selection, all pre-specified, clinically relevant candidate variables were entered into the multivariable models simultaneously using the enter method. This approach was deemed appropriate as our sample size satisfied the recommended criterion of at least 10–15 outcome events per predictor variable, ensuring the stability and reliability of the models. Multicollinearity among the included variables was assessed using variance inflation factors (VIF), with a VIF < 5 considered indicative of acceptable collinearity. Survival curves were generated using the Kaplan–Meier method and compared with the log-rank test. All statistical tests were two-tailed, and a P-value of < 0.05 was considered statistically significant. Analyses were conducted using SPSS software 26.0.

The Cox regression models for recurrence-free survival and overall survival included the following prespecified covariates, entered simultaneously: age, sex, BMI, VFA/lipid group (four levels: Groups 1–4), cardiovascular disease (yes/no), respiratory disease (yes/no), diabetes (yes/no), tumor location (colon/rectum/sigmoid), differentiation grade (low/medium/high), pTNM stage (I/II/III), adjuvant chemotherapy (yes/no), and surgical approach (laparoscopic/open).

## 3 Results

### 3.1 Patient enrollment flowchart analysis

**Figure 2:**
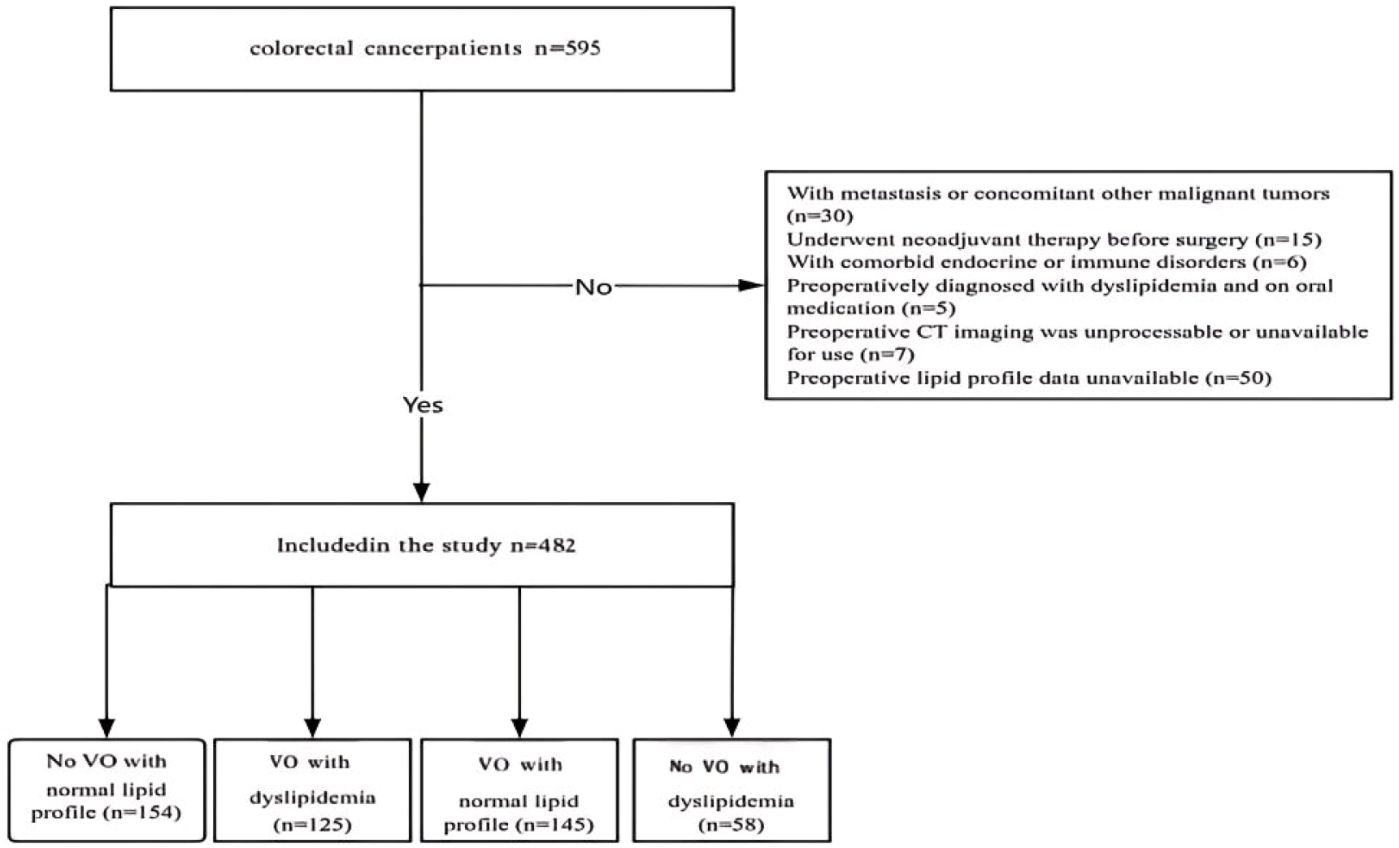
Study enrollment chart. VO, visceral obesity.

This study retrospectively enrolled patients who underwent radical resection for colorectal cancer at Chengdu Second People’s Hospital between December 2014 and December 2022. A total of 482 patients were included and divided into four groups based on visceral fat area (VFA) and blood lipid levels: Group 1 (Low VFA/Normal Lipids, n=154), Group 2 (High VFA/Dyslipidemia, n=125), Group 3 (High VFA/Normal Lipids, n=145), and Group 4 (Low VFA/Dyslipidemia, n=58). **Figure 2** illustrates the enrollment process, from initial screening and exclusion of ineligible patients to final grouping, demonstrating the rigor and transparency of the study.

### 3.2 Comparison of baseline characteristics

The four groups were comparable in most baseline characteristics, including age, sex, and laboratory parameters such as serum albumin, total protein, neutrophil and lymphocyte counts (all *P* > 0.05). Significant differences were observed in several key variables: BMI was highest in Group 2 (24.2, IQR: 22.0–26.0) and lowest in Group 1 (20.4, IQR: 19.1–22.3) (*P* < 0.001); the prevalence of diabetes was highest in Group 2 (28.0%) and lowest in Group 4 (6.9%) (*P* < 0.001); tumor location also differed significantly (*P* = 0.029), with a higher proportion of sigmoid colon tumors in Group 3; operation time and intraoperative blood loss were significantly greater in Groups 2 and 3 (*P* = 0.002 and *P* < 0.001, respectively) (**Table 1**).

**Table 1:** Demographics and baseline characteristics of patients in the four groups.

Characteristic	Group 1 N = 154	Group 2 N = 125	Group 3 N = 145	Group 4 N = 58	Statistic^1^	*P*
Age**, Median (Q1, Q3)**	69 (58, 77)	68 (60, 75)	69 (62, 75)	68 (59, 76)	0.54	0.910
Sex, n (%)					7.18	0.066
**Male**	87 (56.5%)	88 (70.4%)	82 (56.6%)	34 (58.6%)		
**Female**	67 (43.5%)	37 (29.6%)	63 (43.4%)	24 (41.4%)		
**BMI, Median (Q1, Q3)**	20.4 (19.1, 22.3)	24.2 (22.0, 26.0)	23.7 (22.0, 26.0)	20.8 (18.9, 22.7)	144.05	**<0.001**
**Serum Albumin, Median (Q1, Q3)**	40.7 (36.5, 43.3)	41.0 (37.0, 44.3)	41.0 (36.9, 43.9)	39.9 (35.8, 42.5)	3.44	0.329
**Total Protein, Median (Q1, Q3)**	68 (63, 73)	69 (64, 74)	68 (64, 73)	67 (62, 75)	2.96	0.397
**Neutrophils (x10^9^/L), Median (Q1, Q3)**	5.06 (4.37, 5.73)	5.17 (4.42, 5.92)	4.98 (3.95, 5.78)	5.24 (4.42, 5.82)	4.84	0.184
**Lymphocytes (x10^9^/L), Median (Q1, Q3)**	1.67 (1.16, 2.26)	1.59 (1.12, 2.06)	1.63 (1.32, 1.94)	1.76 (1.26, 2.15)	2.35	0.504
**Hemoglobin (g/L), Median (Q1, Q3)**	121 (103, 135)	119 (99, 139)	125 (103, 135)	118 (80, 135)	1.52	0.679
Cardiovascular Disease, n (%)					6.56	0.087
**No**	109 (70.8%)	75 (60.0%)	83 (57.2%)	37 (63.8%)		
**Yes**	45 (29.2%)	50 (40.0%)	62 (42.8%)	21 (36.2%)		
Respiratory Disease, n (%)					2.59	0.459
**No**	112 (72.7%)	101 (80.8%)	112 (77.2%)	45 (77.6%)		
**Yes**	42 (27.3%)	24 (19.2%)	33 (22.8%)	13 (22.4%)		
Diabetes, n (%)					24.99	<0.001
**No**	141 (91.6%)	90 (72.0%)	113 (77.9%)	54 (93.1%)		
**Yes**	13 (8.4%)	35 (28.0%)	32 (22.1%)	4 (6.9%)		
Tumor Location, n (%)					14.06	0.029
**Colon**	36 (23.4%)	33 (26.4%)	28 (19.3%)	20 (34.5%)		
**Rectum**	72 (46.8%)	40 (32.0%)	53 (36.6%)	20 (34.5%)		
**Sigmoid Colon**	46 (29.9%)	52 (41.6%)	64 (44.1%)	18 (31.0%)		
Differentiation Grade, n (%)						
**Low**	16 (10.4%)	19 (15.2%)	17 (11.7%)	12 (20.7%)		
**Medium**	129 (83.8%)	95 (76.0%)	112 (77.2%)	46 (79.3%)		
**High**	9 (5.8%)	11 (8.8%)	16 (11.0%)	0 (0.0%)		
Pathological Stage, n (%)					7.51	0.276
**I**	21 (13.6%)	18 (14.4%)	27 (18.6%)	5 (8.6%)		
**II**	73 (47.4%)	63 (50.4%)	55 (37.9%)	26 (44.8%)		
**III**	60 (39.0%)	44 (35.2%)	63 (43.4%)	27 (46.6%)		
Surgical Approach, n (%)					0.70	0.874
**Open**	55 (35.7%)	44 (35.2%)	46 (31.7%)	21 (36.2%)		
**Laparoscopic**	99 (64.3%)	81 (64.8%)	99 (68.3%)	37 (63.8%)		
ASA Grade, n (%)					3.56	0.313
**<3**	79 (51.3%)	59 (47.2%)	60 (41.4%)	24 (41.4%)		
**≥3**	75 (48.7%)	66 (52.8%)	85 (58.6%)	34 (58.6%)		
**Operation Time (hours), Median (Q1, Q3)**	3.00 (2.50, 4.00)	3.50 (3.00, 4.50)	3.50 (3.00, 4.50)	3.17 (2.92, 4.00)	14.81	**0.002**
**Intraoperative Blood Loss (ml), Median (Q1, Q3)**	50 (50, 100)	80 (50, 100)	80 (50, 100)	50 (50, 100)	16.43	**<0.001**
Prophylactic Stoma, n (%)					5.35	0.148
**No**	132 (85.7%)	107 (85.6%)	125 (86.2%)	43 (74.1%)		
**Yes**	22 (14.3%)	18 (14.4%)	20 (13.8%)	15 (25.9%)		
Postoperative Adjuvant Chemotherapy, n (%)					2.18	0.536
**No**	103 (66.9%)	87 (69.6%)	97 (66.9%)	34 (58.6%)		
**Yes**	51 (33.1%)	38 (30.4%)	48 (33.1%)	24 (41.4%)		

Group 1: low VFA/normal lipids; group 2: high VFA/dyslipidemia; group 3: high VFA/normal lipids; group 4: low VFA/dyslipidemia. Bold values indicate statistical significance (P < 0.05).

### 3.3 Comparison of short-term surgical outcomes

No significant differences were observed among the four groups in terms of overall complications, infectious complications, non-infectious complications, anastomotic leakage, or ileus (*P* > 0.05). Group 2 had the lowest incidence of postoperative hypoproteinemia (6.4%), while Group 1 had the highest (16.2%), approaching statistical significance (*P* = 0.07). Group 3 had the shortest postoperative hospital stay (*P* = 0.02), suggesting a potentially more stable metabolic state (**Table 2**).

**Table 2:** Short-term outcomes in four groups.

Characteristic	Group 1 N = 154	Group 2 N = 125	Group 3 N = 145	Group 4 N = 58	Statistic^1^	*P*
Postoperative Complications, n (%)					0.65	0.884
**No**	110 (71.4%)	93 (74.4%)	104 (71.7%)	40 (69.0%)		
**Yes**	44 (28.6%)	32 (25.6%)	41 (28.3%)	18 (31.0%)		
Postoperative Infectious Complications, n (%)					0.71	0.871
**No**	131 (85.1%)	104 (83.2%)	119 (82.1%)	47 (81.0%)		
**Yes**	23 (14.9%)	21 (16.8%)	26 (17.9%)	11 (19.0%)		
Postoperative Non-infectious Complications, n (%)					1.17	0.760
**No**	123 (79.9%)	106 (84.8%)	119 (82.1%)	47 (81.0%)		
**Yes**	31 (20.1%)	19 (15.2%)	26 (17.9%)	11 (19.0%)		
Postoperative Anastomotic Leakage, n (%)						0.649
**No**	150 (97.4%)	121 (96.8%)	140 (96.6%)	58 (100.0%)		
**Yes**	4 (2.6%)	4 (3.2%)	5 (3.4%)	0 (0.0%)		
Postoperative Hypoproteinemia, n (%)					7.12	0.068
**No**	129 (83.8%)	117 (93.6%)	129 (89.0%)	49 (84.5%)		
**Yes**	25 (16.2%)	8 (6.4%)	16 (11.0%)	9 (15.5%)		
Postoperative Ileus, n (%)						0.232
**No**	152 (98.7%)	118 (94.4%)	140 (96.6%)	56 (96.6%)		
**Yes**	2 (1.3%)	7 (5.6%)	5 (3.4%)	2 (3.4%)		
**FPT (days), Median (Q1, Q3)**	3.00 (2.00, 4.00)	3.00 (2.00, 4.00)	3.00 (2.00, 4.00)	2.00 (2.00, 4.00)	0.43	0.933
**LOS (days), Median (Q1, Q3)**	15 (13, 17)	16 (13, 20)	16 (14, 20)	16 (13, 20)	9.41	**0.024**

FPT, flatus passage time; LOS, length of hospital stay; Group 1: low VFA/normal lipids; group 2: high VFA/dyslipidemia; group 3: high VFA/normal lipids; group 4: lowVFA/dyslipidemia continuous variables (FPT, LOS) were compared using the Kruskal-Wallis test. Categorical variables (complication rates) were compared using the chi-square test or Fisher’s exact test. Bold values indicate statistical significance (P < 0.05)

### 3.4 Univariate and multivariate logistic regression analysis of postoperative complications

**Figure 3:**
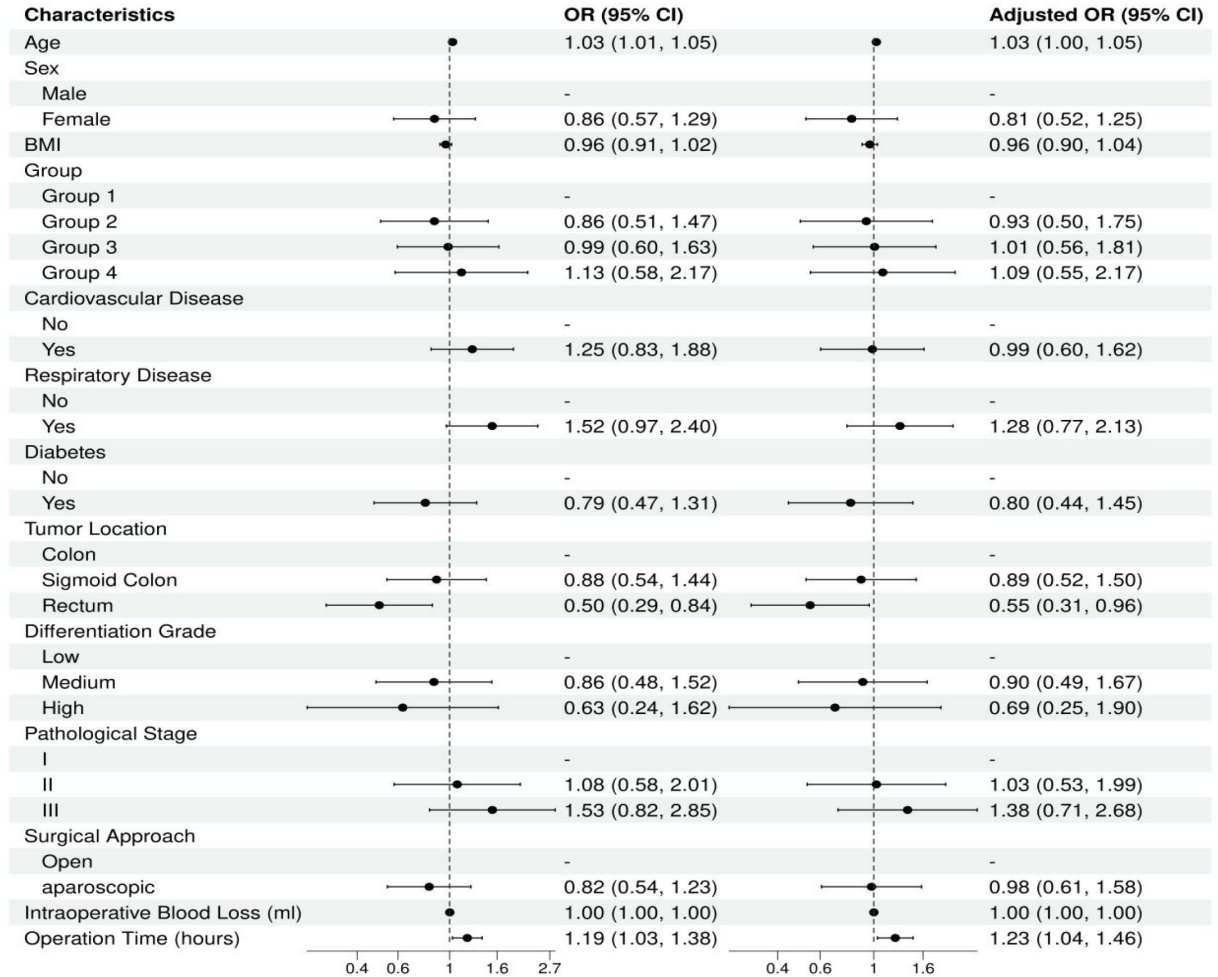
Forest diagram of univariate and multivariate analysis of overall comPlications.

Univariate and multivariate logistic regression analyzes were performed to identify independent factors influencing postoperative complications. Univariate analysis showed that age (OR = 1.03, 95% CI: 1.01–1.05, *P* = 0.002) and operation time (OR = 1.19, 95% CI: 1.03–1.38, *P* = 0.020) were significantly associated with the risk of postoperative complications. Multivariate analysis confirmed that age (aOR = 1.03, 95% CI: 1.00–1.05, *P* = 0.019), sigmoid colon tumor location (compared to other colon locations, aOR = 0.55, 95% CI: 0.31–0.96, *P* = 0.035), and operation time (aOR = 1.23, 95% CI: 1.04–1.46, *P* = 0.018) were independent influencing factors for postoperative complications (**Table 3**). The VFA/lipid group, sex, BMI, comorbidities, tumor differentiation, pathological stage, and surgical approach did not show significant effects in the multivariate model. The corresponding forest plot is shown in **Figure 3**.

**Table 3:** Univariate and multivariate analysis of postoperative overall complications influencing factors (Logistic regression).

Characteristic	Univariable					Multivariable				
	N	Event N	OR	95% CI	*p*	N	Event N	OR	95% CI	*P*
Age	482	135	1.03	1.01, 1.05	0.002	482	135	1.03	1.00, 1.05	0.019
Sex										
Female	191	57	—	—		191	57	—	—	
Male	291	78	0.86	0.57, 1.29	0.468	291	78	0.81	0.52, 1.25	0.345
BMI	482	135	0.96	0.91, 1.02	0.209	482	135	0.96	0.90, 1.04	0.316
Group (Ref: croup 1)										
Group 1	154	44	—	—		154	44	—	—	
Group 2	125	32	0.86	0.51, 1.47	0.579	125	32	0.93	0.50, 1.75	0.826
Group 3	145	41	0.99	0.60, 1.63	0.955	145	41	1.01	0.56, 1.81	0.979
Group 4	58	18	1.13	0.58, 2.17	0.725	58	18	1.09	0.55, 2.17	0.806
Cardiovascular disease										
No	304	80	—	—		304	80	—	—	
Yes	178	55	1.25	0.83, 1.88	0.280	178	55	0.99	0.60, 1.62	0.958
Respiratory disease										
No	370	96	—	—		370	96	—	—	
Yes	112	39	1.52	0.97, 2.40	0.068	112	39	1.28	0.77, 2.13	0.331
Diabetes										
No	398	108	—	—		398	108	—	—	
Yes	84	27	1.27	0.76, 2.11	0.354	84	27	1.25	0.69, 2.26	0.467
Tumor location (Ref: colon)										
Colon	117	40	—	—		117	40	—	—	
Rectum	185	58	0.88	0.54, 1.44	0.608	185	58	0.89	0.52, 1.50	0.653
Sigmoid Colon	180	37	0.50	0.29, 0.84	0.009	180	37	0.55	0.31, 0.96	0.035
Differentiation grade (Ref: low)										
Low	64	20	—	—		64	20	—	—	
Medium	382	107	0.86	0.48, 1.52	0.595	382	107	0.90	0.49, 1.67	0.740
High	36	8	0.63	0.24, 1.62	0.337	36	8	0.69	0.25, 1.90	0.473
Pathological stage (Ref: I)										
I	71	17	—	—		71	17	—	—	
II	217	55	1.08	0.58, 2.01	0.813	217	55	1.03	0.53, 1.99	0.940
III	194	63	1.53	0.82, 2.85	0.182	194	63	1.38	0.71, 2.68	0.340
Surgical approach										
Open	166	51	—	—		166	51	—	—	
Laparoscopic	316	84	0.82	0.54, 1.23	0.336	316	84	0.98	0.61, 1.58	0.930
Intraoperative Blood Loss (ml)	482	135	1.00	1.00, 1.00	0.141	482	135	1.00	1.00, 1.00	0.402
Operation Time (hours)	482	135	1.19	1.03, 1.38	0.020	482	135	1.23	1.04, 1.46	0.018

Group 1: low VFA/normal lipids; group 2: high VFA/dyslipidemia; group 3: high VFA/normal lipids; group 4: low VFA/dyslipidemia OR, odds ratio; CI, confidence interval; BMI, body mass index; ref, reference category. For logistic regression models, P values were derived from the likelihood ratio test. Bold values indicate statistical significance (P < 0.05).

### 3.5 Univariate and multivariate Cox regression analysis of recurrence-free survival

Univariate analysis showed that age, pathological stage, postoperative adjuvant chemotherapy, and surgical approach were significantly associated with recurrence-free survival (RFS). Multivariate Cox regression analysis indicated that age was an independent risk factor for recurrence, with a 3% increase in recurrence risk per year (aHR = 1.03, 95% CI: 1.01–1.04, *P* = 0.002). Compared to Group 1 (low VFA with normal lipids), Group 2 (High VFA/Dyslipidemia) was a protective factor against recurrence (aHR = 0.50, 95% CI: 0.30–0.85, *P* = 0.009). History of cardiovascular disease was also a protective factor (aHR = 0.68, 95% CI: 0.47–0.99, *P* = 0.045). Pathological stage III was a strong risk factor, significantly increasing recurrence risk compared to stage I (aHR = 5.68, 95% CI: 2.72–11.86, *P* < 0.001). Laparoscopic surgery was a protective factor compared to open surgery (aHR = 0.64, 95% CI: 0.45–0.89, *P* = 0.009). Postoperative adjuvant chemotherapy was associated with an increased risk of recurrence (aHR = 1.52, 95% CI: 1.07–2.17, *P* = 0.020) (**Table 4**).

**Table 4:** Univariate and multivariate analysis of RFS influencing factors (Cox regression).

Characteristic	Univariable					Multivariable				
	N	Event N	HR	95% CI	*P*	N	Event N	HR	95% CI	*p*
Age	482	164	1.02	1.00, 1.03	0.016	482	164	1.03	1.01, 1.04	0.002
Sex										
Female	191	61	—	—		191	61	—	—	
Male	291	103	1.15	0.84, 1.58	0.384	291	103	1.34	0.96, 1.88	0.088
BMI	482	164	0.99	0.95, 1.04	0.732	482	164	1.03	0.98, 1.09	0.241
Group (Ref: group 1)										
Group 1	154	51	—	—		154	51	—	—	
Group 2	125	31	0.66	0.42, 1.04	0.072	125	31	0.50	0.30, 0.85	0.009
Group 3	145	58	1.21	0.83, 1.77	0.316	145	58	0.99	0.65, 1.53	0.974
Group 4	58	24	1.46	0.90, 2.38	0.124	58	24	1.43	0.86, 2.37	0.164
Cardiovascular disease										
No	304	109	—	—		304	109	—	—	
Yes	178	55	0.83	0.60, 1.15	0.268	178	55	0.68	0.47, 0.99	0.045
Respiratory disease										
No	370	122	—	—		370	122	—	—	
Yes	112	42	1.27	0.89, 1.80	0.185	112	42	1.00	0.68, 1.46	0.985
Diabetes										
No	398	136	—	—		398	136	—	—	
Yes	84	28	0.94	0.63, 1.42	0.781	84	28	1.30	0.83, 2.05	0.257
Tumor location (Ref: colon)										
Colon	117	37	—	—		117	37	—	—	
Rectum	185	57	0.94	0.62, 1.42	0.758	185	57	1.03	0.67, 1.60	0.884
Sigmoid Colon	180	70	1.18	0.79, 1.76	0.410	180	70	1.42	0.92, 2.17	0.109
Differentiation grade (Ref: low)										
Low	64	28	—	—		64	28	—	—	
Medium	382	128	0.66	0.44, 1.00	0.049	382	128	0.76	0.50, 1.15	0.191
High	36	8	0.40	0.18, 0.88	0.022	36	8	0.66	0.30, 1.49	0.321
Pathological stage (Ref: I)										
I	71	8	—	—		71	8	—	—	
II	217	53	2.40	1.14, 5.04	0.021	217	53	2.17	1.02, 4.61	0.043
III	194	103	6.62	3.22, 13.60	<0.001	194	103	5.68	2.72, 11.86	<0.001
Postoperative adjuvant chemotherapy										
No	321	94	—	—		321	94	—	—	
Yes	161	70	1.69	1.24, 2.30	<0.001	161	70	1.52	1.07, 2.17	0.020
Surgical approach										
Open	166	68	—	—		166	68	—	—	
Laparoscopic	316	96	0.67	0.49, 0.92	0.012	316	96	0.64	0.45, 0.89	0.009

Group 1: Low VFA/Normal Lipids;Group 2: High VFA/Dyslipidemia;Group 3: High VFA/Normal Lipids;Group 4: Low VFA/Dyslipidemia Adjusted covariates in the multivariable Cox model: age, sex, BMI, VFA/lipid group (four levels), cardiovascular disease, respiratory disease, diabetes, tumor location, differentiation grade, pTNM stage, adjuvant chemotherapy, and surgical approach. For Cox regression models, P values were derived from the Wald test.Bold values indicate statistical significance (P < 0.05).

### 3.6 Univariate and multivariate Cox regression analysis of overall survival

Univariate analysis indicated that age, VFA/lipid group, history of respiratory disease, pathological stage, surgical approach, and prophylactic stoma were significantly associated with overall survival (OS). Multivariate Cox regression analysis showed that increasing age was an independent risk factor for death, with a 3% increase in mortality risk per year (aHR = 1.03, 95% CI: 1.02–1.05, *P* < 0.001). Compared to Group 1 (low VFA with normal lipids), Group 2 (High VFA/Dyslipidemia) was a protective factor (aHR = 0.49, 95% CI: 0.30–0.81, *P* = 0.005). Pathological stage III was an independent risk factor, significantly increasing mortality risk compared to stage I (aHR = 2.96, 95% CI: 1.62–5.43, *P* < 0.001). Laparoscopic surgery was associated with reduced mortality risk compared to open surgery (aHR = 0.68, 95% CI: 0.49–0.94, *P* = 0.021). Prophylactic stoma was a risk factor, significantly associated with increased mortality risk (aHR = 1.82, 95% CI: 1.26–2.64, *P* = 0.001). Additionally, sigmoid colon tumors showed a trend towards increased mortality risk compared to other colon locations, though this did not reach statistical significance (aHR = 1.50, 95% CI: 0.98–2.28, *P* = 0.060) (**Table 5**).

**Table 5:** Univariate and multivariate analysis of OS influencing factors (Cox regression).

Characteristic	Univariable					Multivariable				
	N	Event N	HR	95% CI	*p*	N	Event N	HR	95% CI	*p*
Age	482	173	1.03	1.02, 1.04	<0.001	482	173	1.03	1.02, 1.05	<0.001
Sex										
Female	191	65	—	—		191	65	—	—	
Male	291	108	1.13	0.83, 1.54	0.428	291	108	1.22	0.88, 1.69	0.224
BMI	482	173	0.98	0.94, 1.02	0.270	482	173	1.04	0.98, 1.10	0.162
Group (Ref: group 1)										
Group 1	154	57	—	—		154	57	—	—	
Group 2	125	31	0.61	0.39, 0.94	0.025	125	31	0.49	0.30, 0.81	0.005
Group 3	145	59	1.09	0.76, 1.57	0.646	145	59	0.88	0.58, 1.33	0.535
Group 4	58	26	1.38	0.87, 2.20	0.172	58	26	1.35	0.84, 2.18	0.214
Cardiovascular disease										
No	304	110	—	—		304	110	—	—	
Yes	178	63	0.97	0.71, 1.32	0.841	178	63	0.79	0.55, 1.12	0.189
Respiratory disease										
No	370	122	—	—		370	122	—	—	
Yes	112	51	1.51	1.09, 2.10	0.013	112	51	1.15	0.81, 1.63	0.441
Diabetes										
No	398	143	—	—		398	143	—	—	
Yes	84	30	0.99	0.67, 1.47	0.964	84	30	1.21	0.78, 1.87	0.394
Tumor location (Ref: colon)										
Colon	117	39	—	—		117	39	—	—	
Rectum	185	59	0.93	0.62, 1.39	0.710	185	59	0.95	0.61, 1.46	0.802
Sigmoid Colon	180	75	1.26	0.86, 1.86	0.236	180	75	1.50	0.98, 2.28	0.060
Differentiation grade (Ref: low)										
Low	64	24	—	—		64	24	—	—	
Medium	382	137	0.82	0.53, 1.27	0.375	382	137	0.85	0.54, 1.32	0.460
High	36	12	0.73	0.36, 1.46	0.371	36	12	1.07	0.52, 2.20	0.853
Pathological stage (Ref: I)										
I	71	13	—	—		71	13	—	—	
II	217	65	1.76	0.97, 3.19	0.063	217	65	1.52	0.83, 2.80	0.177
III	194	95	3.40	1.90, 6.06	<0.001	194	95	2.96	1.62, 5.43	<0.001
Postoperative adjuvant chemotherapy										
No	321	114	—	—		321	114	—	—	
Yes	161	59	1.09	0.80, 1.50	0.575	161	59	1.12	0.79, 1.60	0.528
Surgical approach										
Open	166	70	—	—		166	70	—	—	
Laparoscopic	316	103	0.69	0.51, 0.93	0.017	316	103	0.68	0.49, 0.94	0.021

Group 1: low VFA/normal lipids; group 2: high VFA/dyslipidemia; group 3: high VFA/normal lipids; group 4: low VFA/dyslipidemia adjusted covariates in the multivariable cox model: age, sex, BMI, VFA/lipid group (four levels), cardiovascular disease, respiratory disease, diabetes, tumor location, differentiation grade, pTNM stage, adjuvant chemotherapy, and surgical approach. For Cox regression models, P values were derived from the Wald test.Bold values indicate statistical significance (P < 0.05).

### 3.7 Survival curve analysis

**Figure 4:**
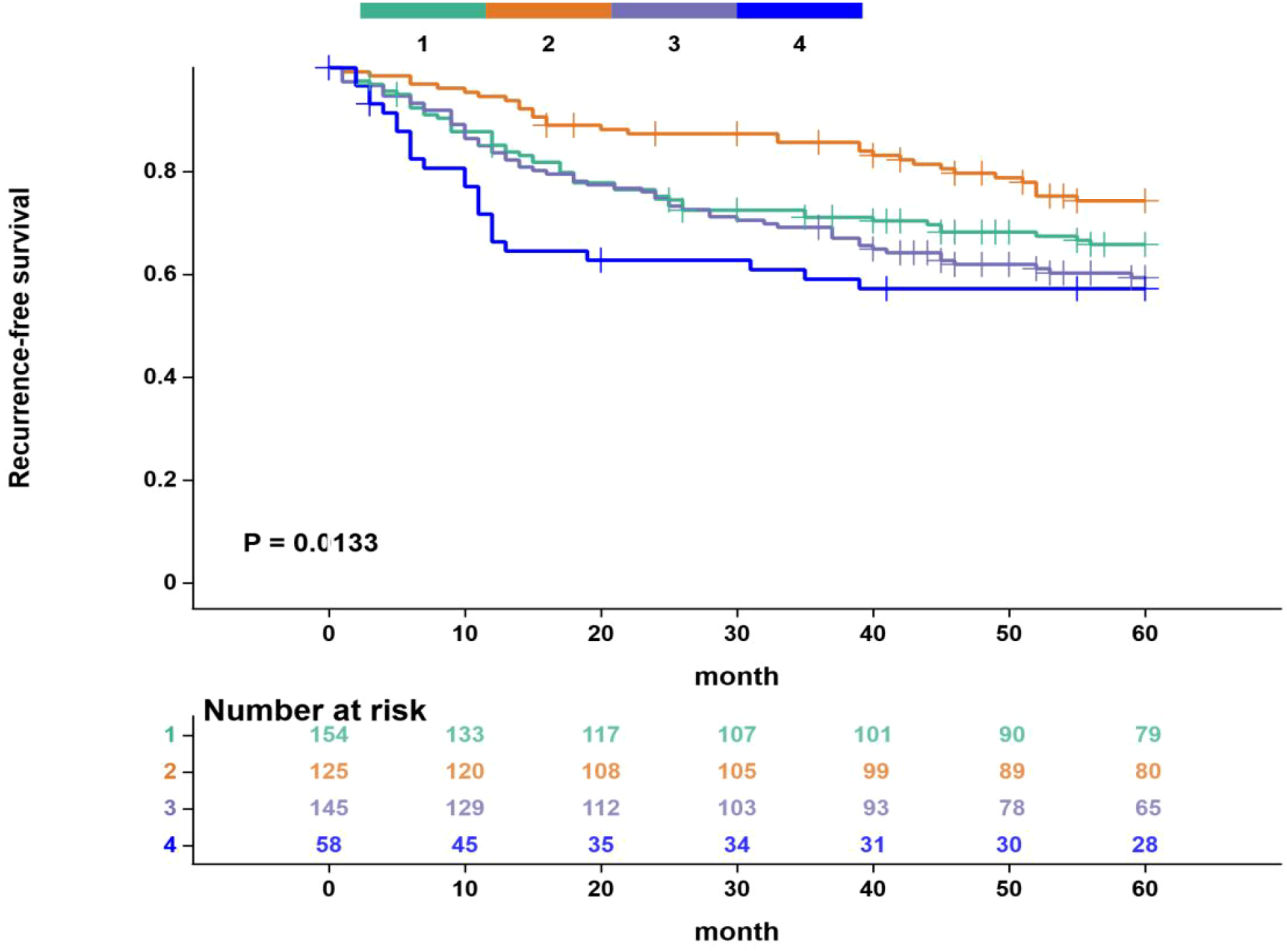
Kaplan-Meier curves for recurrence-free survival based on four groups. Group 1: Low VFA/Normal Lipids;Group 2: High VFA/Dyslipidemia;Group 3: High VFA/Normal Lipids;Group 4: Low VFA/Dyslipidemia The maximum follow-up period was 60 months. Censored patients are indicated by tick marks. Numbers at risk are shown below the x-axis.Survival curves were compared using the log-rank test (overall P < 0.05).

**Figure 5:**
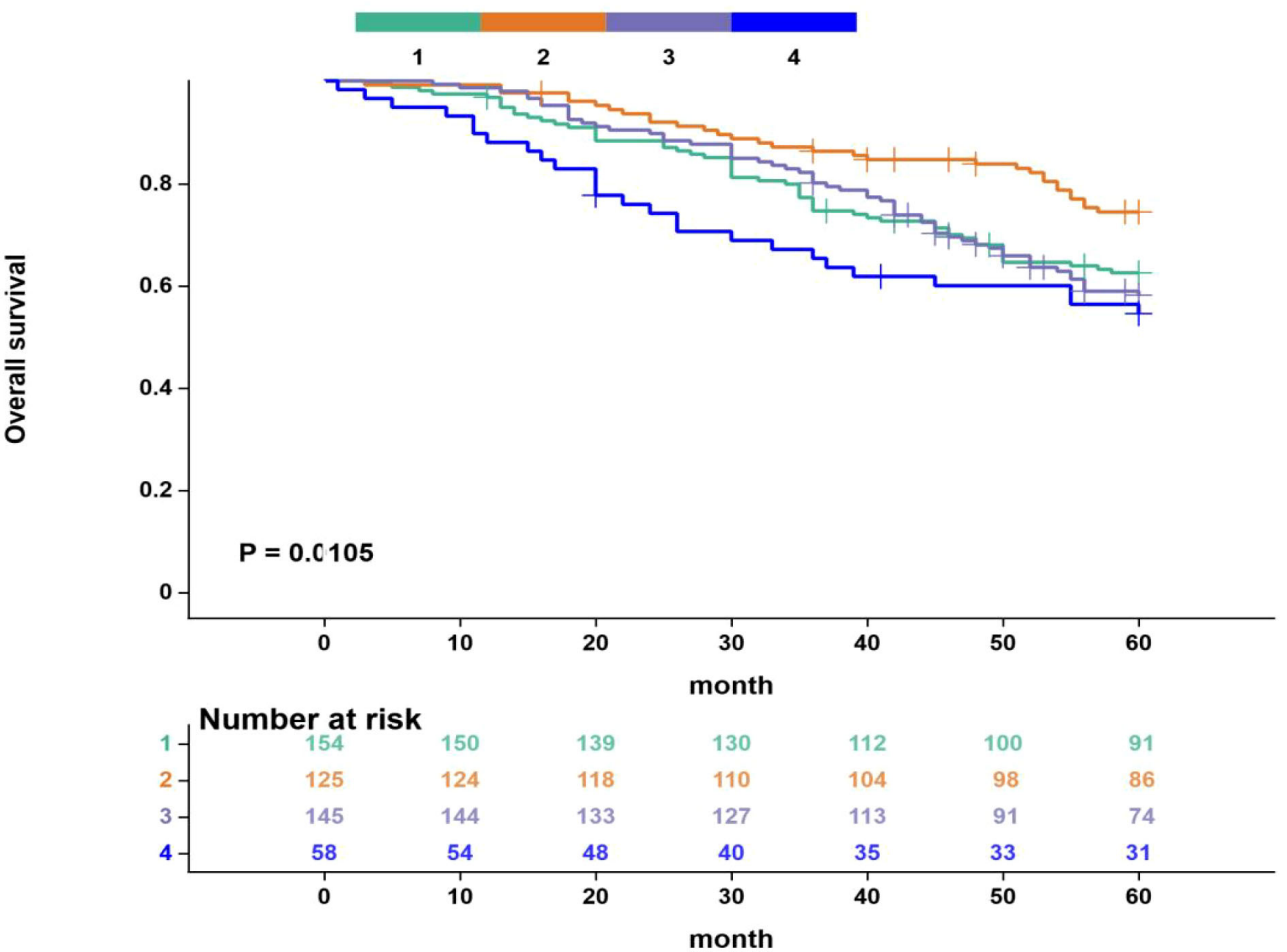
Kaplan-Meier curves for overall survival based on four groups. Group 1: Low VFA/Normal Lipids;Group 2: High VFA/Dyslipidemia;Group 3: High VFA/Normal Lipids;Group 4: Low VFA/Dyslipidemia The maximum follow-up period was 60 months. Censored patients are indicated by tick marks. Numbers at risk are shown below the x-axis.Survival curves were compared using the log-rank test (overall P < 0.05).

Kaplan-Meier survival analysis revealed significant differences in recurrence-free survival (RFS) and overall survival (OS) among the four subgroups defined by VFA and blood lipid levels. Specifically, the RFS and OS curves for Group 2 (High VFA/Dyslipidemia) were consistently positioned highest, indicating the most favorable survival prognosis. The survival curves for Group 4 (Low VFA/Dyslipidemia) were positioned lowest, suggesting a significantly worse prognosis compared to the other groups. The survival outcomes for Group 1 (Low VFA/Normal Lipids) and Group 3 (High VFA/Normal Lipids) were intermediate. These results further support the use of the combined VFA and lipid indicator as an effective tool for prognostic stratification (**Figures 4**, **5**).

## 4 Discussion

This study represents the first systematic evaluation of the combined predictive value of visceral fat area (VFA) and blood lipid levels for both perioperative risk and long-term prognosis in colorectal cancer (CRC) patients undergoing radical resection. Through a retrospective analysis of 482 patients, we demonstrated that the composite VFA/lipid index had limited utility in predicting short-term postoperative complications but showed significant value in stratifying long-term survival outcomes. Notably, patients with both high VFA and high blood lipid levels (Group 2) exhibited the most favorable overall survival (OS) and recurrence-free survival (RFS), with a hazard ratio (HR) as low as 0.50. This finding provides compelling evidence supporting the existence of the “obesity paradox” in the context of CRC.

### 4.1 Association of the VFA/lipid profile with short-term surgical outcomes

No statistically significant differences were observed among the four patient groups—stratified by VFA and blood lipid levels—in terms of overall complications, infectious complications, or non-infectious complications (*P* > 0.05). Multivariate logistic regression identified advanced age (adjusted odds ratio [aOR] = 1.03), longer operation time (aOR = 1.23), and tumor location in the sigmoid colon (aOR = 0.55) as independent predictors of postoperative complications. In contrast, the VFA/lipid grouping was not an independent risk factor.

Several factors may explain these neutral findings regarding short-term outcomes. First, the influence of metabolic abnormalities may be overshadowed by more immediate surgical and anesthetic factors (20), such as intraoperative trauma, hemodynamic instability, and physiological stress responses. These acute factors likely dominate the early postoperative period, outweighing subtler metabolic contributions.

Second, the pathophysiological effects of visceral obesity and dyslipidemia are generally chronic and systemic (21), mediated through mechanisms such as sustained low-grade inflammation, insulin resistance, and immune modulation (22, 23). These processes are unlikely to exert acute effects sufficient to alter surgical risk within a short timeframe.

Moreover, although Group 2 patients presented with a higher metabolic burden, they also demonstrated significantly better nutritional reserve, as reflected by the lowest incidence of postoperative hypoproteinemia (6.4%). This nutritional advantage may have counterbalanced the potential risks associated with obesity and dyslipidemia, thereby mitigating disparities in short-term surgical outcomes across groups.

### 4.2 Potential mechanisms and alternative explanations for the observed association

The observed “obesity paradox”—where patients with high VFA and high lipid levels showed the best long-term survival—can be interpreted through several interrelated biological mechanisms (24–26).

#### 4.2.1 Nutritional reserve hypothesis

(27): Adequate energy stores in the form of visceral adipose tissue may provide a crucial buffer during the catabolic state induced by both cancer and its treatments. This reserve can help maintain physiological homeostasis, support immune function, and improve tolerance to chemotherapy and surgery. In our cohort, Group 2 patients had the lowest rate of hypoproteinemia, underscoring their superior nutritional status and metabolic resilience.

#### 4.2.2 Inflammatory and immune modulation

(28, 29): Obesity is typically associated with chronic low-grade inflammation, which is generally considered detrimental. However, our data revealed that Group 2 patients had lower neutrophil counts, higher lymphocyte counts, and a more favorable neutrophil-to-lymphocyte ratio (NLR) compared to other groups. This profile suggests a moderated inflammatory response and preserved immune competence. A balanced inflammatory milieu may hinder tumor proliferation and metastasis, thereby conferring a survival advantage (27, 30).

#### 4.2.3 Metabolic reprogramming

(31–33): Elevated lipid levels in conjunction with high visceral adiposity may alter tumor metabolism. Lipids serve as key energy substrates and signaling molecules, potentially influencing cancer cell behavior. Although not directly measured in this study, specific lipid species or derivatives may exert anti-tumor effects, or alternatively, the hyperlipidemic environment might induce metabolic dependencies that can be therapeutically exploited. The interplay between lipid availability, adipokine secretion, and tumor metabolism warrants further investigation.

While we propose several biological mechanisms, alternative non-causal explanations must be considered. First, reverse causation is possible: patients with more aggressive tumors may experience cancer-related weight loss and lipid depletion (i.e., cachexia), leading to lower VFA and lipid levels (Group 1 or Group 4), rather than low adiposity per se causing worse outcomes. Second, residual confounding by smoking, physical activity, dietary patterns, or socioeconomic status—variables not collected in this retrospective study—cannot be excluded. Third, selection bias may have occurred if patients with poor performance status or severe comorbidities were disproportionately excluded. Therefore, our findings should be interpreted as supporting an association and risk stratification value, not as evidence that hyperlipidemia or high VFA is directly beneficial.

### 4.3 Interpretation of the worst prognostic phenotype: low VFA with high lipids

Patients in Group 4 (Low VFA/Dyslipidemia) demonstrated the poorest survival outcomes. This phenotype may represent a metabolically dysfunctional state, often associated with sarcopenia and systemic metabolic dysregulation (34–36). Unlike nutritional obesity, high lipid levels in this context may reflect pathological conditions such as insulin resistance, hepatogenic lipid dysmetabolism, or para-inflammatory responses secondary to tumor progression (36–38).

This group may be experiencing early cancer cachexia, wherein metabolic disarray—rather than nutritional excess—drives poor outcomes (34, 35, 37). The combination of low energy reserves and dyslipidemia (39, 40) likely indicates compromised adaptive capacity, accompanied by chronic inflammation and immune exhaustion. These factors collectively accelerate tumor progression and increase mortality (41). Clinicians should recognize this “high-risk metabolic phenotype” and consider early nutritional and metabolic interventions.

### 4.4 Adjuvant chemotherapy and recurrence: confounding by indication

In our multivariate Cox regression analysis, postoperative adjuvant chemotherapy was unexpectedly associated with an increased risk of recurrence (aHR = 1.52, 95% CI: 1.07–2.17,*P* = 0.020). We emphasize that this association should not be interpreted as a harmful effect of chemotherapy. Rather, it almost certainly reflects confounding by indication—a well-recognized bias in observational studies of treatment effects. Specifically, patients with higher-risk tumor features (e.g., advanced pTNM stage, lymphovascular invasion, or positive margins) are more likely to receive adjuvant chemotherapy and simultaneously have a higher baseline risk of recurrence. Although we adjusted for pTNM stage in the model, residual or unmeasured confounding (e.g., tumor grade, molecular subtypes, or patient performance status) cannot be fully excluded. Therefore, no causal conclusion regarding the effect of adjuvant chemotherapy on recurrence can be drawn from these data. This finding serves as a reminder that associations observed in retrospective studies may be driven by treatment selection biases rather than true biological effects, and it does not challenge the established clinical benefit of adjuvant chemotherapy in high-risk colorectal cancer patients.

### 4.5 Limitations and future directions

This study has several limitations. Its single-center, retrospective nature introduces potential selection bias. Our definition of dyslipidemia represents a single-time-point, untreated laboratory phenotype, which may not fully capture the dynamic or treated dyslipidemia status in real-world clinical practice. Although VFA quantification was standardized, it still entails a degree of operator-dependent variability.

Future research should involve multi-center prospective designs with repeated measurements of metabolic and inflammatory markers (42–44)—such as dynamic lipid profiles, NLR, platelet-to-lymphocyte ratio (PLR), and prognostic nutritional index (PNI). Body composition analysis, including skeletal muscle area and density, should be integrated to evaluate sarcopenia. Molecular subtyping (45–47) (e.g., Consensus Molecular Subtypes [CMS]) may further reveal interactions between metabolic profiles and tumor biology.

Additionally, exploring specific lipid metabolites and inflammatory cytokines could (42) help decipher the mechanistic links between body composition, metabolic health, and cancer outcomes.

### 4.6 Clinical and translational implications

The VFA-lipid composite index is an accessible preoperative tool that can enhance risk stratification and personalize management in CRC patients. For Group 2 patients (High VFA/Dyslipidemia), although perioperative risks should be managed cautiously, their favorable long-term prognosis may justify more aggressive antitumor therapies coupled with continuous metabolic monitoring and support.

Conversely, Group 4 patients (Low VFA/Dyslipidemia) require early identification and multidisciplinary management involving nutritional support, physical conditioning, metabolic correction, and close surveillance. Tailored interventions aimed at reversing catabolism and modulating inflammation may improve outcomes in this high-risk subgroup.

This metabolic stratification complements traditional TNM staging and may serve as a pragmatic adjunct in clinical decision-making, may aid preoperative stratification and perioperative metabolic/nutritional assessment; prospective validation is warranted.

### 4.7 Conclusion

In summary, the composite VFA-lipid index stratifies colorectal cancer patients into subgroups with distinct long-term survival, independent of pathological stage. Patients with high VFA and dyslipidemia (Group 2) had the best prognosis, while those with low VFA and dyslipidemia (Group 4) had the worst. These associations may involve nutritional and inflammatory factors, although reverse causation and residual confounding cannot be excluded.

This index is readily available from routine preoperative CT and blood tests and may complement TNM staging for risk assessment. However, given the retrospective, single-center design, our findings are hypothesis-generating. Prospective, multi-center validation is needed before clinical implementation.

## Data Availability

The raw data supporting the conclusions of this article will be made available by the authors, without undue reservation.
