# Supplementary material for: Visceral fat area and blood lipids in colorectal cancer: predictors of surgical risk and prognosis
Source: Front Oncol. 2026 May 4;16:1809966. doi: 10.3389/fonc.2026.1809966 (PMC13180551; doi:10.3389/fonc.2026.1809966)
Supplement: Supplementary file 1 [file DataSheet1.docx]

# **Supplementary Material**

****Title:**Visceral Fat Area and Blood Lipids in Colorectal Cancer: Predictors of Surgical Risk and Prognosis**

## Supplementary Methods

### S1. Definition of Dyslipidemia for Patient Grouping

To address the potential inconsistency between the exclusion criterion (history of diagnosed dyslipidemia) and the grouping variable, we clarify that the term “Dyslipidemia” used for patient stratification refers strictly to **laboratory-defined dyslipidemia** based on preoperative fasting blood samples, regardless of prior diagnosis. Patients with a documented history of dyslipidemia who were receiving lipid-lowering therapy were excluded (exclusion criterion #5). Therefore, the “Dyslipidemia” groups (Group 2 and Group 4) consist of patients who met any of the following laboratory thresholds without ongoing pharmacological treatment: total cholesterol (TC) ≥ 6.2 mmol/L, low-density lipoprotein cholesterol (LDL-C) ≥ 4.1 mmol/L, triglycerides (TG) ≥ 2.3 mmol/L, or high-density lipoprotein cholesterol (HDL-C) < 1.0 mmol/L. This definition reflects an **untreated baseline lipid phenotype**.

### S2. Data Derivation Statement for Patient Grouping

Patients were classified into four groups based on two binary criteria:

Visceral obesity (VO): defined as visceral fat area (VFA) ≥ 100 cm² measured at the L3 vertebral level.

Dyslipidemia : defined as meeting any one of the following laboratory thresholds (preoperative fasting blood sample):

TC ≥ 6.2 mmol/L, or LDL-C ≥ 4.1 mmol/L, or TG ≥ 2.3 mmol/L, or HDL-C < 1.0 mmol/L.

The four groups were then derived as follows:

Group 1 (Low VFA/Normal Lipids): VFA < 100 cm² AND no dyslipidemia.

Group 2 (High VFA/Dyslipidemia): VFA ≥ 100 cm² AND dyslipidemia present.

Group 3 (High VFA/Normal Lipids): VFA ≥ 100 cm² AND no dyslipidemia.

Group 4 (Low VFA/Dyslipidemia): VFA < 100 cm² AND dyslipidemia present.

This derivation was applied to all 482 patients prior to any statistical analysis. No ambiguous or missing assignments occurred.

### S3. Proportional Hazards Assumption Testing

The proportional hazards (PH) assumption for all Cox regression models was tested using Schoenfeld residuals. No significant violations were detected (global test *P* > 0.05 for all final models). Log-minus-log survival plots were also visually inspected and supported the PH assumption.

### S4. Follow-Up and Censoring

## The median follow-up time for the overall survival endpoint was calculated using the reverse Kaplan‑Meier method and was 60 months (interquartile range [IQR]: 40–60 months). The minimum follow-up was 1 month and the maximum was 60 months. During the follow-up period, death (the outcome event) was observed in 173 patients (35.9%), and censoring occurred in 309 patients (64.1%) due to loss to follow-up or being alive at the end of the study.

## Regarding postoperative complications (within 30 days after surgery), a total of 135 patients (28.0%) experienced any complication. Among these, infectious complications occurred in 81 patients (16.8%), and non‑infectious complications in 87 patients (18.0%). The most frequent specific complications were hypoproteinemia (58 patients, 12.0%), followed by postoperative ileus (16 patients, 3.3%) and anastomotic leakage (13 patients, 2.7%).

## Recurrence was ascertained by contrast‑enhanced CT or PET‑CT and/or histopathological biopsy according to institutional protocols.

## Supplementary Results

### S1. Descriptive Statistics of Lipid Variables

**Table S1** presents the descriptive statistics of the four lipid variables in the overall cohort $\text{N}\text{=482}$. **Table S2** shows their distribution after stratification into quartiles.

**Table S1. Descriptive statistics of lipid variables**

| Variable | Mean | SD | Minimum | Maximum | N |
| --- | --- | --- | --- | --- | --- |
| TG (mmol/L) | 1.37 | 0.89 | 0.31 | 11.21 | 482 |
| TC (mmol/L) | 4.12 | 1.05 | 0.96 | 7.70 | 482 |
| HDL-C (mmol/L) | 1.18 | 0.36 | 0.37 | 3.21 | 482 |
| LDL-C (mmol/L) | 2.35 | 0.77 | 0.58 | 5.17 | 482 |

*Abbreviations:*SD, standard deviation; TG, triglycerides; TC, total cholesterol; HDL-C, high-density lipoprotein cholesterol; LDL-C, low-density lipoprotein cholesterol.

**Table S2. Quartile distribution of lipid variables**

| Variable | Quartile | Mean | SD | Range | N |
| --- | --- | --- | --- | --- | --- |
| TG | Q1 | 0.72 | 0.14 | 0.31–0.89 | 125 |
|  | Q2 | 1.03 | 0.08 | 0.90–1.15 | 118 |
|  | Q3 | 1.33 | 0.11 | 1.16–1.54 | 118 |
|  | Q4 | 2.42 | 1.21 | 1.55–11.21 | 121 |
| TC | Q1 | 2.83 | 0.47 | 0.96–3.33 | 121 |
|  | Q2 | 3.75 | 0.20 | 3.35–4.07 | 122 |
|  | Q3 | 4.45 | 0.22 | 4.08–4.85 | 120 |
|  | Q4 | 5.49 | 0.59 | 4.86–7.70 | 119 |
| HDL-C | Q1 | 0.80 | 0.11 | 0.37–0.94 | 126 |
|  | Q2 | 1.04 | 0.05 | 0.95–1.13 | 117 |
|  | Q3 | 1.25 | 0.07 | 1.14–1.37 | 118 |
|  | Q4 | 1.65 | 0.29 | 1.38–3.21 | 121 |
| LDL-C | Q1 | 1.43 | 0.29 | 0.58–1.80 | 122 |
|  | Q2 | 2.08 | 0.14 | 1.81–2.34 | 123 |
|  | Q3 | 2.56 | 0.14 | 2.35–2.80 | 116 |
|  | Q4 | 3.35 | 0.48 | 2.81–5.17 | 121 |

*Abbreviations:SD, standard deviation; TG, triglycerides; TC, total cholesterol; HDL-C, high-density lipoprotein cholesterol; LDL-C, low-density lipoprotein cholesterol.*

### S2. Continuous Lipid Variables and Outcomes

When modeled as continuous variables (adjusted for age, sex, BMI, and pTNM stage), TG was significantly associated with 5-year recurrence (HR = 0.763, 95% CI: 0.596–0.978, *P* = 0.0326), and TC was significantly associated with postoperative infectious complications (OR = 0.726, 95% CI: 0.568–0.928, *P* = 0.0107). No other significant associations were observed for continuous lipid variables with overall complications, infectious complications, recurrence, or mortality (all *P* > 0.05).

### S3. Threshold Effects of Lipid Variables on Infectious Complications

When lipid variables were modeled as quartiles (Q1 as reference), significant threshold effects were observed for TC and LDL-C on postoperative infectious complications, but not for TG or HDL-C. **Table S3** summarizes these findings.

**Table S3. Threshold effects (quartile analysis) for postoperative infectious complications – only significant findings**

| Variable | Comparison | OR (95% CI) | *P* | *P* for trend |
| --- | --- | --- | --- | --- |
| TC | Q4 vs. Q1 | 0.367 (0.173–0.780) | 0.009 | 0.0116 |
| LDL-C | Q4 vs. Q1 | 0.428 (0.207–0.885) | 0.022 | 0.0174 |

*Abbreviations:Adjusted for age, sex, BMI, and pTNM stage. No significant threshold effects were observed for TG or HDL-C, nor for overall complications, 5-year recurrence, or 5-year mortality (all P for trend > 0.05). Abbreviations as in Tables S1 and S3.*

### S4. Stratified Cox Regression Analysis by pTNM Stage

**Table S4** presents the results of stratified Cox regression analyses for recurrence-free survival (RFS) and overall survival (OS) according to pTNM stage (I, II, or III), using the same covariate set as the main models. The protective effect of Group 2 (High VFA/Dyslipidemia) remained consistent across stages.

Table S4. Stratified Cox regression analysis for RFS and OS by pTNM stage (I/II/III)

| Variable | RFS |  |  | OS |  |  |
| --- | --- | --- | --- | --- | --- | --- |
|  | z | *p* | HR (95% CI) | z | *p* | HR (95% CI) |
| Age | 3.12 | 0.001 | 1.03 (1.01–1.04) | 3.76 | 0.001 | 1.03 (1.02–1.05) |
| BMI | 1.17 | 0.24 | 1.03 (0.98–1.09) | 1.1 | 0.27 | 1.03 (0.98–1.09) |
| Group |  |  |  |  |  |  |
| Group 1 (ref) | – | – | – | – | – | – |
| Group 2 | -2.59 | 0.01 | 0.51 (0.30–0.85) | -2.79 | 0.01 | 0.49 (0.30–0.81) |
| Group 3 | -0.05 | 0.96 | 0.99 (0.64–1.52) | -0.69 | 0.49 | 0.87 (0.57–1.31) |
| Group 4 | 1.3 | 0.19 | 1.40 (0.84–2.31) | 1.39 | 0.17 | 1.40 (0.87–2.25) |
| Sex |  |  |  |  |  |  |
| Female (ref) | – | – | – | – | – | – |
| Male | -1.7 | 0.09 | 0.75 (0.53–1.05) | -1.52 | 0.13 | 0.78 (0.56–1.07) |
| Cardiovascular disease |  |  |  |  |  |  |
| No (ref) | – | – | – | – | – | – |
| Yes | -1.97 | 0.05 | 0.69 (0.48–1.00) | -1.32 | 0.19 | 0.79 (0.55–1.12) |
| Respiratory disease |  |  |  |  |  |  |
| No (ref) | – | – | – | – | – | – |
| Yes | -0.05 | 0.96 | 0.99 (0.67–1.45) | 0.93 | 0.35 | 1.18 (0.83–1.68) |
| Diabetes |  |  |  |  |  |  |
| No (ref) | – | – | – | – | – | – |
| Yes | 1.12 | 0.26 | 1.30 (0.82–2.04) | 1.08 | 0.28 | 1.27 (0.82–1.97) |
| Tumour location |  |  |  |  |  |  |
| colon (ref) | – | – | – | – | – | – |
| **Sigmoid Colon** | 1.59 | 0.11 | 1.41 (0.92–2.16) | 2.43 | 0.02 | 1.67 (1.10–2.52) |
| **Rectum** | 0.16 | 0.88 | 1.04 (0.67–1.60) | 0.12 | 0.9 | 1.03 (0.67–1.57) |
| Differentiation |  |  |  |  |  |  |
| Moderate (ref) | – | – | – | – | – | – |
| Poor | 1.31 | 0.19 | 1.32 (0.87–2.02) | 0.76 | 0.45 | 1.19 (0.76–1.85) |
| High | -0.37 | 0.71 | 0.87 (0.42–1.81) | 0.67 | 0.5 | 1.23 (0.67–2.27) |
| Adjuvant chemotherapy |  |  |  |  |  |  |
| No (ref) | – | – | – | – | – | – |
| Yes | 2.32 | 0.02 | 1.52 (1.07–2.16) | 0.51 | 0.61 | 1.10 (0.77–1.56) |
| Surgical approach |  |  |  |  |  |  |
| Open (ref) | – | – | – | – | – | – |
| Laparoscopic | -2.53 | 0.01 | 0.65 (0.46–0.91) | -2.32 | 0.02 | 0.68 (0.49–0.94) |

*Abbreviations: HR, hazard ratio; CI, confidence interval; BMI, body mass index; pTNM, pathological tumor-node-metastasis. Group definitions: Group 1 (Low VFA/Normal Lipids), Group 2 (High VFA/Dyslipidemia), Group 3 (High VFA/Normal Lipids), Group 4 (Low VFA/Dyslipidemia).*
